# Entropic Dynamics on Gibbs Statistical Manifolds

**DOI:** 10.3390/e23050494

**Published:** 2021-04-21

**Authors:** Pedro Pessoa, Felipe Xavier Costa, Ariel Caticha

**Affiliations:** Department of Physics, University at Albany (SUNY), Albany, NY 12222, USA

**Keywords:** entropic dynamics, maximum entropy, information geometry, canonical distributions, exponential family

## Abstract

Entropic dynamics is a framework in which the laws of dynamics are derived as an application of entropic methods of inference. Its successes include the derivation of quantum mechanics and quantum field theory from probabilistic principles. Here, we develop the entropic dynamics of a system, the state of which is described by a probability distribution. Thus, the dynamics unfolds on a statistical manifold that is automatically endowed by a metric structure provided by information geometry. The curvature of the manifold has a significant influence. We focus our dynamics on the statistical manifold of Gibbs distributions (also known as canonical distributions or the exponential family). The model includes an “entropic” notion of time that is tailored to the system under study; the system is its own clock. As one might expect that entropic time is intrinsically directional; there is a natural arrow of time that is led by entropic considerations. As illustrative examples, we discuss dynamics on a space of Gaussians and the discrete three-state system.

## 1. Introduction

The original method of Maximum Entropy (MaxEnt) is usually associated with the names of Shannon [1] and Jaynes [2,3,4,5], although its roots can be traced to Gibbs [6]. The method was designed to assign probabilities on the basis of partial information in the form of expected value constraints and the central quantity, called entropy, which was interpreted as a measure of uncertainty or as an amount of missing information. In a series of developments starting with Shore and Johnson [7], with further contributions from other authors [8,9,10,11,12], the range of applicability of the method was significantly extended. In its new incarnation, the purpose of the method of Maximum Entropy, which will be referred as ME to distinguish it from the older version, is to update the probabilities from arbitrary priors when new information in the form of constraints is considered [13]. Highlights of the new method include: (1) A unified treatment of Bayesian and entropic methods which demonstrates their mutual consistency. (2) A new concept of entropy as a tool for reasoning that requires no interpretation in terms of heat, multiplicities, disorder, uncertainty, or amount of information. Indeed, entropy in ME needs no interpretation; it is a tool designed to perform a certain function—to update probabilities to accommodate new information. (3) A Bayesian concept of information defined in terms of its effects on the beliefs of rational agents—the constraints are the information. (4) The possibility of information that is not in the form of expected value constraints (we shall see an example below).

The old MaxEnt was sufficiently versatile for providing the foundations to equilibrium statistical mechanics [2] and to find application in a wide variety of fields such as economics [14], ecology [15,16], cellular biology [17,18], network science [19,20], and opinion dynamics [21,22]. As is the case with thermodynamics, all these applications are essentially static. MaxEnt has also been deployed to non-equilibrium statistical mechanics (see [23,24] and subsequent literature in maximum caliber, e.g., [25,26,27]) but the dynamics is not intrinsic to the probabilities; it is induced by the underlying Hamiltonian dynamics of the molecules. For problems beyond physics there is a need for more general dynamical frameworks based on information theory.

The ME version of the maximum entropy method offers the possibility of developing a true dynamics of probabilities. It is a dynamics driven by entropy—an Entropic Dynamics (ED)—which is automatically consistent with the principles for updating probabilities. ED naturally leads to an “entropic” notion of time. Entropic time is a device designed to keep track of the accumulation of changes. Its construction involves three ingredients: one must introduce the notion of an instant, verify that these instants are suitably ordered, and finally one must define a convenient notion of duration or interval between successive instants. A welcome feature is that entropic time is tailored to the system under study; the system is its own clock. Another welcome feature is that such an entropic time is intrinsically directional—an arrow of time is generated automatically.

ED has been successful in reconstructing dynamical models in physics such as quantum mechanics [28,29], quantum field theory [30], and the renormalization group [31]. Beyond physics, it has been recently applied to the rhw fields of finance [32,33] and neural networks [34]. Here, we aim for a different class of applications of ED: to describe the dynamics of Gibbs distributions, also known as canonical distribution (exponential family) in statistical physics (statistics), since they are the distributions that are defined by a set of expected values constraint, namely sufficient statistics. Unlike the other cited papers on ED, here we will not focus on what the distributions are meant to represent. Other assumptions that would be specific to the modeled system are beyond the scope of the present article.

The goal is to study the ED that is generated by transitions from one distribution to another. The main assumptions are that changes happen and that they are not discontinuous. We do not explain why changes happen—this is a mechanics without a mechanism. Our goal is to venture an educated estimate of what changes one expects to happen. The second assumption is that systems evolve along continuous trajectories in the space of probability distributions. It also implies that the study of motion involves two tasks. The first is to describe how a single infinitesimal step occurs. The second requires a scheme for keeping track of how a large number of these short steps accumulate to produce a finite motion. It is the latter task that involves the introduction of the concept of time.

The fact that the space of macrostates is a statistical manifold—each point in the space is a probability distribution—has a profound effect on the dynamics. The reason is that statistical manifolds are naturally endowed with a Riemannian metric structure that is given by the Fisher–Rao information metric (FRIM) [35,36]; this structure is known as information geometry [37,38,39]. The particular case of Gibbs distributions leads to additional interesting geometrical properties (see e.g., [40,41]), which have been explored in the extensive work relating statistical mechanics to information geometry [42,43,44,45,46,47,48,49]. Information geometry has also been used as a fundamental concept for complexity measures [50,51,52].

In this paper, we tackle the more formal aspects of an ED on Gibbs manifolds and offer a couple of illustrative examples. The formalism is applied to two important sets of probability distributions: the space of Gaussians and the space of distributions for a three-state system, both of which can be written in the exponential form. Because these distributions are both well-studied and scientifically relevant, they can give us a good insight into how the dynamics work.

It is important to emphasize that Gibbs distributions are not restricted to the description of a system in thermal equilibrium. While it is true that, if one chooses the conserved quantities in Hamiltonian motion as the sufficient statistics, the resultant Gibbs distributions are the ones that are associated to equilibrium statistical mechanics, the Gibbs distribution can be defined for arbitrary choices of sufficient statistics, and the modeling endeavour includes identifying the ones that are relevant to the problem at hand. On the same note, the dynamics developed here are not a form of nonequilibrium statistical mechanics, which is driven by a underlying physical molecular dynamics, while the ED is completely agnostic of any microstate dynamics.

The article is organized, as follows: the next section discusses the space of Gibbs distributions and its geometric properties; Section 3 considers the ideas of ED; Section 4 tackles the difficulties associated with formulating ED on the curved space of probability distributions; Section 5 introduces the notion of entropic time; Section 6 describes the evolution of the system in the form of a differential equation; in Section 7, we offer two illustrative examples of ED on a Gaussian manifold and on a two-simplex.

## 2. The Statistical Manifold of Gibbs Distributions

### 2.1. Gibbs Distributions

The canonical or Gibbs probability distributions are the macrostates of a system. They describe a state of uncertainty regarding the microstate x∈X of the macroscopic system. A canonical distribution ρ(x) is assigned by maximizing the entropy

(1) $$S\left[\rho |q\right]=-\int dx\;\rho \left(x\right)\log\frac{\rho (x)}{q(x)}$$

relative to the prior q(x) subject to *n* expected value constraints

(2) $$\int dx\;\rho \left(x\right)a^{i}\left(x\right)=A^{i},\;\mathrm{with}\;i=1\ldots n\;,$$

and the normalization of ρ(x). Typically, the prior q(x) is chosen to be a uniform distribution over the space X so that it is maximally non-informative, but this is not strictly necessary. The *n* constraints, on the other hand, reflect the information that happens to be relevant to the problem. The resulting canonical distribution is

(3) $$\rho \left(x|\lambda \right)=\frac{q(x)}{Z(\lambda )}\exp\left[-\lambda_{i}a^{i}\left(x\right)\right],$$

where $\lambda =\{\lambda_{1}\ldots \lambda_{n}\}$ are the Lagrange multipliers that are associated to the expected value constraints, and we adopt the Einstein summation convention. The normalization constant is

(4) $$Z\left(\lambda \right)=\int dx\;q\left(x\right)\exp\left[-\lambda_{i}a^{i}\left(x\right)\right]=e^{-F(\lambda )},$$

where F=−logZ plays a role analogous to the free energy. The Lagrange multipliers $\lambda_{i}\left(A\right)$ are implicitly defined by

(5) $$\frac{\partial F}{\partial \lambda_{i}}=A^{i}\;.$$

Evaluating the entropy (1) at its maximum yields

(6) $$S\left(A\right)=-\int dx\;\rho \left(x|\lambda \left(A\right)\right)\log\frac{\rho (x|\lambda (A))}{q(x)}=\lambda_{i}\left(A\right)A^{i}-F\left(\lambda \left(A\right)\right)\;.$$

which we shall call the macrostate entropy or (when there is no risk of confusion) just the entropy. Equation (6) shows that S(A) is the Legendre transform of F(λ): a small change $dA^{i}$ in the constraints shows that S(A) is indeed a function of the expected values $A^{i}$,

(7) $$dS=\lambda_{i}dA^{i}\;\mathrm{so}\;\mathrm{that}\;\lambda_{i}=\frac{\partial S}{\partial A^{i}}\;.$$

One might think that defining dynamics on the family of canonical distributions might be too restricted to be of interest; however, this family has widespread applicability. Here, it has been derived using the method of maximum entropy, but historically it has also been known as the exponential family, namely the only family of distributions that possesses sufficient statistics. Interestingly, this was a problem that was proposed by Fisher [53] in the primordium of statistics and later proved independently by Pitman [54], Darmois [55], and Koopman [56]. The sufficient statistics turn out to be the functions $a_{i}\left(x\right)$ in (1). In Table 1, we give a short list of the priors q(x) and the functions $a_{i}\left(x\right)$ that lead to well-known distributions [41,57].

Naturally, the method of maximum entropy assumes that the various constraints are compatible with each other, so that the set of multipliers λ exists. It is further assumed that the constraints reflect physically relevant information, so that the various functions, such as $A^{i}\left(\lambda \right)=\frac{\partial }{\partial \lambda_{i}}F$ and $\lambda_{i}\left(A\right)=\frac{\partial }{\partial A^{i}}S$, which appear in the formalism, are both invertible and differentiable, and so that the space of Gibbs distributions is indeed a manifold. However, the manifold may include singularities of various kinds that are of particular interest, as they may describe phenomena, such as phase transitions [42,46].

### 2.2. Information Geometry

We offer a brief review of well known results concerning the information geometry of Gibbs distributions in order to establish the notation and recall some results that will be needed in later sections [38,40].

To each set of expected values $A=\{A^{1},A^{2},\ldots ,A^{n}\}$, or to the associated set of Lagrange multipliers $\lambda =\{\lambda_{1},\lambda_{2},\ldots ,\lambda_{n}\}$, there corresponds a canonical distribution. Therefore the set of distributions ρ(x|λ) or, equivalently, ρ(x|A) is a statistical manifold in which each point can be labelled by the coordinates λ or by *A*. Whether we choose λ or *A* as coordinates is purely a matter of convenience. The change of coordinates is implemented using

(8) $$\frac{\partial A^{i}}{\partial \lambda_{k}}=-\frac{\partial^{2}\log Z}{\partial \lambda_{k}\partial \lambda_{i}}=A^{k}A^{i}-\langle a^{k}a^{i}\rangle \;,$$

where we recognize the covariance tensor,

(9) $$C^{ij}=\langle \left(a^{i}-A^{i}\right)\left(a^{j}-A^{j}\right)\rangle =-\frac{\partial A^{i}}{\partial \lambda_{j}}\;.$$

Its inverse is given by

(10) $$C_{jk}=-\frac{\partial \lambda_{j}}{\partial A^{k}}=-\frac{\partial^{2}S}{\partial A^{j}\partial A^{k}}\;,$$

that means the inverse covariant matrix $C_{ij}$ is the Hessian of negative entropy in (6). This implies

(11) $$C^{ij}C_{jk}=\frac{\partial A^{i}}{\partial A^{k}}=\delta_{k}^{i}\;.$$

Statistical manifolds are endowed with an essentially unique quantity to measure the extent to which two neighboring distributions ρ(x|A) and ρ(x|A+dA) can be distinguished from each other. This measure of distinguishability provides a statistical notion of distance, which is given by FRIM, $d\ell^{2}=g_{ij}dA^{i}dA^{j}$ where

(12) $$g_{ij}=\int dx\;\rho \left(x|A\right)\frac{\partial \log\rho (x|A)}{\partial A^{i}}\frac{\partial \log\rho (x|A)}{\partial A^{j}}.$$

For a broader discussion on the existence, derivation, and consistency of this metric, as well as its properties, see [38,39,40]. Here, it suffices to say that FRIM is the unique metric structure that is invariant under Markov embeddings [58,59] and, therefore, is the only way of assigning a differential geometry structure that is in accordance to the grouping property of probability distributions.

To calculate $g_{ij}$ for canonical distributions, we use

(13) $$g_{ij}=\frac{\partial \lambda_{k}}{\partial A^{i}}\frac{\partial \lambda_{l}}{\partial A^{j}}\int dx\;\rho \;\frac{\partial \log\rho }{\partial \lambda_{k}}\;\frac{\partial \log\rho }{\partial \lambda_{l}}$$

and

(14) $$\frac{\partial \log\rho (x|A)}{\partial \lambda_{k}}=A^{k}-a^{k}\left(x\right)$$

so that, using (8)–(11), we have

(15) $$g_{ij}=C_{ik}C_{lj}C^{kl}=C_{ij}\;.$$

Therefore, the metric tensor $g_{ij}$ is the inverse of the covariance matrix $C^{ij}$, which, by (10), is the Hessian of the entropy.

As mentioned above, instead of $A^{i}$, we could use the Lagrange multipliers $\lambda_{i}$ as coordinates. Subsequently, the information metric is the covariance matrix,

(16) $$g^{ij}=\int dx\;\rho \left(x|\lambda \right)\;\frac{\partial \log\rho (x|\lambda )}{\partial \lambda_{i}}\;\frac{\partial \log\rho (x|\lambda )}{\partial \lambda_{j}}=C^{ij}\;.$$

Therefore, the distance dℓ between neighboring distributions can be written in either of two equivalent forms,

(17) $$d\ell^{2}=g_{ij}dA^{i}dA^{j}=g^{ij}d\lambda_{i}d\lambda_{j}\;.$$

Incidentally, the availability of a unique measure of volume $dV={\left(\det g_{ij}\right)}^{1/2}d^{n}A$ implies that there is a uniquely defined notion of the uniform distribution over the space of macrostates. The uniform distribution $P_{u}$ assigns equal probabilities to equal volumes, so that

(18) $$P_{u}\left(A\right)d^{n}A\propto g^{1/2}d^{n}A\;\mathrm{where}\;g=\det g_{ij}\;.$$

To conclude this overview section, we note that the metric tensor $g_{ij}$ can be used to lower the contravariant indices of a vector to produce its dual covector. Using (10) and (12), the covector $dA_{i}$ dual to the infinitesimal vector with components $dA^{i}$ is

(19) $$dA_{i}=g_{ij}dA^{j}=-\frac{\partial \lambda_{i}}{\partial A^{j}}dA^{j}=-d\lambda_{i}\;.$$

which shows that not only are the coordinates *A* and λ related through a Legendre transformation, which is a consequence of entropy maximization, but also through a vector-covector duality, i.e., $-d\lambda_{i}$ is the covector dual to $dA^{i}$, which is a consequence of information geometry.

## 3. Entropic Dynamics

Having established the necessary background, we can now develop an entropic framework to describe the dynamics on the space of macrostates.

### 3.1. Change Happens

Our starting assumption is that changes happen continuously, which is supported by observation in nature. Therefore, the dynamics that we wish to formulate assumes that the system evolves along continuous paths. This assumption of continuity represents a significant simplification, because it implies that a finite motion can be analyzed as the accumulation of a large number of infinitesimally short steps. Thus, our first goal will be to find the probability $P(A^{\prime }|A)$ that the system takes a short step from the macrostate *A* to the neighboring macrostate $A^{\prime }=A+dA$. The transition probability $P(A^{\prime }|A)$ will be assigned by maximizing an entropy. This first requires that we identify the particular entropy that is relevant to our problem. Next, we must decide on the prior distribution: what short steps we might expect before we know the specifics of the motion. Finally, we stipulate the constraints that are meant to capture the information that is relevant to the particular problem at hand.

To settle the first item—the choice of entropy—we note that not only are we are uncertain about the macrostate at *A*, but we are also uncertain about the microstates x∈X. This means that the actual universe of discourse is the joint space A×X and the appropriate statistical description of the system is in terms of the joint distribution

(20) P(x,A)=P(x|A)P(A)=ρ(x|A)P(A),

Where ρ is of form (3), which means that we impose P(x|A) to be canonical and the distribution P(A) represents our lack of knowledge about the macrostates. Note that what we did in (20) is nothing more than assuming a probability distribution for the macrostates. This description is sometimes referred to as superstatistics [60].

Our immediate task is to find the transition probability of a change $P(x^{\prime },A^{\prime }|x,A)$ by maximizing the entropy

(21) $$S\left[P|Q\right]=-\int dA^{\prime }dx^{\prime }P\left(x^{\prime },A^{\prime }|x,A\right)\log\frac{P(x^{\prime },A^{\prime }|x,A)}{Q(x^{\prime },A^{\prime }|x,A)}\;,$$

relative to the prior $Q(x^{\prime },A^{\prime }|x,A)$ and subject to constraints to be discussed below (to simplify the notation in multidimensional integrals we write $d^{n}A^{\prime }=dA^{\prime }$ and $d^{n}x^{\prime }=dx^{\prime }$).

Although *S* in (6) and S in (21) are both entropies, in the information theory sense, they represent two very distinct statistical objects. The S(A) in (6) is the entropy of the macrostate—which is what one may be used to from statistical mechanics —while the S[P|Q] in (21) is the entropy to be maximized, so that we find the transition probability that better matches the information at hand, which means that S is a tool to select the dynamics of the macrostates.

### 3.2. The Prior

We adopt a prior that implements the idea that the system evolves by taking short steps A→A+ΔA at the macrostate level, but is otherwise maximally uninformative. We write

(22) $$Q\left(x^{\prime },A^{\prime }|x,A\right)=Q\left(x^{\prime }|x,A,A^{\prime }\right)Q\left(A^{\prime }|x,A\right)\;,$$

and analyze the two factors in turn. We shall assume that a priori, before we know the relation between the microstates *x* and the macrostate *A*, the prior distribution for $x^{\prime }$ is the same uniform underlying measure $q(x^{\prime })$ that is introduced in (1),

(23) $$Q\left(x^{\prime }|x,A,A^{\prime }\right)=q\left(x^{\prime }\right)\;.$$

Next, we tackle the second factor $Q(A^{\prime }|x,A)$. As shown in Appendix A, using the method of maximum entropy, the prior that enforces short steps, but is otherwise maximally uninformative, is spherically symmetric as

(24) $$Q\left(A^{\prime }|x,A\right)=Q\left(A^{\prime }|A\right)\propto g^{1/2}\left(A^{\prime }\right)\exp\left[-\frac{1}{2\tau }g_{ij}\Delta A^{i}\Delta A^{j}\right]\;.$$

so the joint prior is

(25) $$Q\left(x^{\prime },A^{\prime }|x,A\right)\propto q\left(x^{\prime }\right)g^{1/2}\left(A^{\prime }\right)\exp\left[-\frac{1}{2\tau }g_{ij}\Delta A^{i}\Delta A^{j}\right]\;.$$

We see that steps of length

(26) $$\Delta \ell ∼{\left(g_{ij}\Delta A^{i}\Delta A^{j}\right)}^{1/2}\gg \tau^{1/2}\;,$$

have negligible probability. Eventually, we will take the limit τ→0 to enforce short steps. The prefactor $g^{1/2}\left(A^{\prime }\right)$ ensures that $Q(A^{\prime }|A)$ is a probability density. Later, we will show how this choice of priors, which only comes from the assumption of continuous motion, leads to a diffusion structure.

### 3.3. The Constraints

The piece of information that we wish to codify through the constraints is the simple geometric idea that the dynamics remains confined to the statistical manifold A. This is implemented by writing

(27) $$P\left(x^{\prime },A^{\prime }|x,A\right)=P\left(x^{\prime }|x,A,A^{\prime }\right)P\left(A^{\prime }|x,A\right)$$

and imposing that the distribution for $x^{\prime }$ is a canonical distribution

(28) $$P\left(x^{\prime }|x,A,A^{\prime }\right)=\rho \left(x^{\prime }|A^{\prime }\right)\in A\;.$$

This means that, given $A^{\prime }$, the distribution of $x^{\prime }$ is independent of the initial microstate *x* and macrostate *A*. The second factor in (27), $P(A^{\prime }|x,A)$, is the transition probability we seek, which leads to the constraint

(29) $$P\left(x^{\prime },A^{\prime }|x,A\right)=\rho \left(x^{\prime }|A^{\prime }\right)P\left(A^{\prime }|x,A\right).$$

We note that this constraint is not, as is usual in applications of the method of maximum entropy, in the form of an expected value. It may appear from (29) that the transition probability $P(A^{\prime }|x,A)$ will be largely unaffected by the underlying space of microstates. To the contrary, as we shall see below—(31) and (32)—the macrostate dynamics turns out to be dominated by the entropy of the microstate distribution $\rho (x^{\prime }|A^{\prime })$.

Depending on the particular system under consideration, one could formulate richer forms of dynamics by imposing additional constraints. To give just one example, one could introduce some drift relative to the direction that is specified by a covector $F_{i}$ by imposing a constraint of the form $\langle \Delta A^{i}\rangle F_{i}=\kappa$ (see [29,30]). However, in this paper, we shall limit ourselves to what is perhaps the simplest case, the minimal ED that is described by the single constraint (29).

### 3.4. Maximizing the Entropy

Substituting (25) and (29) into (21) and rearranging, we find

(30) $$S\left[P|Q\right]=\int dA^{\prime }\;P\left(A^{\prime }|x,A\right)\left[-\log\frac{P(A^{\prime }|x,A)}{Q(A^{\prime }|A)}+S\left(A^{\prime }\right)\right]$$

where $S(A^{\prime })$ is the macrostate entropy that is given in (6). Maximizing S subject to normalization gives

(31) $$\begin{array}{cc} P(A^{\prime }|x,A) & \propto Q\left(A^{\prime }|A\right)e^{S(A^{\prime })}\\ \; & \propto g^{1/2}\left(A^{\prime }\right)\exp\left[-\frac{1}{2\tau }g_{ij}\Delta A^{i}\Delta A^{j}+S\left(A^{\prime }\right)\right]\;. \end{array}$$

It is noteworthy that $P(A^{\prime }|x,A)$ turned out to be independent of *x*, which is not surprising, since neither the prior nor the constraints indicate any correlation between $A^{\prime }$ and *x*.

We perform a linear approximation of *S* because the transition from *A* to $A^{\prime }$ has to be an arbitrarily small continuous change. This makes the exponential factor in (31) quadratic in ΔA, as

(32) $$P\left(A^{\prime }|A\right)=\frac{g^{1/2}\left(A^{\prime }\right)}{Z}\exp\left[\frac{\partial S}{\partial A^{i}}\Delta A^{i}-\frac{1}{2\tau }g_{ij}\Delta A^{i}\Delta A^{j}\right],$$

where $e^{S(A)}$ was absorbed in the normalization factor Z. This is the transition probability found by maximizing the entropy (21). However, some mathematical difficulties arise from the fact that (32) is defined over a curved manifold. We are going to explore these mathematical issues and their consequences to motion in the following section.

## 4. The Transition Probability

Because the statistical manifold is a curved space, we must understand how the transition probability (32) behaves under a change of coordinates. Because (25) and (32) describe an arbitrarily small step, we wish to express the transition probability, as well as the quantities derived from it, which are calculated up to the order of τ. Because the exponent in (32) is manifestly invariant, one can complete squares and obtain

(33) $$P\left(A^{\prime }|A\right)=\frac{g^{1/2}\left(A^{\prime }\right)}{Z^{\prime }}\exp\left[-\frac{1}{2\tau }g_{ij}\left(\Delta A^{i}-\tau g^{ik}\frac{\partial S}{\partial A^{k}}\right)\left(\Delta A^{j}-\tau g^{ik}\frac{\partial S}{\partial A^{k}}\right)\right]\;.$$

If g(A) were uniform, it would imply that the first two moments $\Delta A^{i}$ and $\Delta A^{i}\Delta A^{j}$ are of order τ. Therefore, even in the limit τ→0, the transition will be affected by curvature effects. This can be verified for an arbitrary metric tensor by a direct calculation of the first moment,

(34) $$\begin{array}{cc} \langle \Delta A^{i}\rangle = & \int dA^{\prime }\;\Delta A^{i}P\left(A^{\prime }|A\right)\\ = & \frac{1}{Z^{\prime }}\int dA^{\prime }\;g^{1/2}\left(A^{\prime }\right)\Delta A^{i}\exp\left[-\frac{g_{kl}}{2\tau }\left(\Delta A^{k}-\tau V^{k}\right)\left(\Delta A^{l}-\tau V^{l}\right)\right]\;, \end{array}$$

where $V^{i}=g^{ij}\frac{\partial S}{\partial A^{j}}\;$. And the second moment

(35) $$\begin{array}{cc} \langle \Delta A^{i}\Delta A^{j}\rangle = & \int dA^{\prime }\;\Delta A^{i}\Delta A^{j}P\left(A^{\prime }|A\right)\\ = & \frac{1}{Z^{\prime }}\int dA^{\prime }\;g^{1/2}\left(A^{\prime }\right)\Delta A^{i}\Delta A^{j}\exp\left[-\frac{g_{kl}}{2\tau }\left(\Delta A^{k}-\tau V^{k}\right)\left(\Delta A^{l}-\tau V^{l}\right)\right]\;. \end{array}$$

It is convenient to write (32) in normal coordinates at *A* in order to facilitate the calculation of the integrals in (34) and (35). This means that, for a smooth manifold, one can always make a change of coordinates $A^{\mu }\left(A^{i}\right)$—we will label the normal coordinates with Greek letter indexes (μ,ν)—so that the metric in this coordinate system is so that

(36) $$g_{\mu \nu }\left(A\right)=\delta_{\mu \nu }\;\;\mathrm{and}\;\;\frac{\partial g_{\mu \nu }}{\partial A^{\mu }}{|}_{A}=0\;,$$

allowing for us to approximate $g(A^{\prime })=1$ for a short step. For a general discussion and rigorous proof of the existence of normal coordinates, see [61]. Although normal coordinates are a valuable tool for geometrical analysis at this point, it is not clear whether they can be given a deeper statistical interpretation—this is unlike other applications of differential geometry, such as general relativity, where the physical interpretation of normal coordinates turns out be of central importance. A displacement in these coordinates $\Delta A^{\mu }$ can be related to the original coordinates by a Taylor expansion in terms of $\Delta A^{i}$ as (see [62,63])

(37) $$\Delta A^{\mu }=\frac{\partial A^{\mu }}{\partial A^{i}}\Delta A^{i}+\frac{1}{2}\frac{\partial^{2}A^{\mu }}{\partial A^{j}\partial A^{k}}\Delta A^{j}\Delta A^{k}+o\left(\tau \right)\;.$$

To proceed, it is interesting to recall the Christoffel symbols $\Gamma_{jk}^{i}$,

(38) $$\Gamma_{jk}^{i}=\frac{1}{2}g^{il}\left(\frac{\partial g_{jl}}{\partial A^{l}}+\frac{\partial g_{lj}}{\partial A^{k}}-\frac{\partial g_{jk}}{\partial A^{l}}\right)\;,$$

which transform as

(39) $$\Gamma_{jk}^{i}=\frac{\partial A^{i}}{\partial A^{\mu }}\frac{\partial A^{\nu }}{\partial A^{j}}\frac{\partial A^{\sigma }}{\partial A^{k}}\Gamma_{\nu \sigma }^{\mu }-\frac{\partial A^{i}}{\partial A^{\mu }}\frac{\partial^{2}A^{\mu }}{\partial A^{j}\partial A^{k}}\;.$$

Because, in normal coordinates, we have $\Gamma_{\nu \sigma }^{\mu }=0$, this allows us to isolate $\Delta A^{i}$ up to the order τ obtaining

(40) $$\Delta A^{i}=\frac{\partial A^{i}}{\partial A^{\mu }}\Delta A^{\mu }-\frac{1}{2}\Gamma_{jk}^{i}\Delta A^{j}\Delta A^{k}\;,$$

By squaring (40), we have

(41) $$\Delta A^{i}\Delta A^{j}=\frac{\partial A^{i}}{\partial A^{\mu }}\frac{\partial A^{j}}{\partial A^{\nu }}\Delta A^{\mu }\Delta A^{\nu }+o\left(\tau \right)\;.$$

Because the exponent in (34) is invariant and in a coordinate transformation we have $dA\;P\left(A\right)=d\tilde{A}\;P\left(\tilde{A}\right)$, it separates into two terms.

(42) $$\begin{array}{cc} \langle \Delta A^{i}\rangle & =\frac{\partial A^{i}}{\partial A^{\mu }}\frac{1}{Z^{\prime }}\int dA^{\prime }\Delta A^{\mu }\exp\left[-\frac{\delta_{\nu \sigma }}{2\tau }\left(\Delta A^{\nu }-\tau V^{\nu }\right)\left(\Delta A^{\sigma }-\tau V^{\sigma }\right)\;\right]\\ \; & -\frac{1}{2}\Gamma_{jk}^{i}\frac{\partial A^{j}}{\partial A^{\mu }}\frac{\partial A^{k}}{\partial A^{\nu }}\frac{1}{Z^{\prime }}\int dA^{\prime }\Delta A^{\mu }\Delta A^{\nu }\exp\left[-\frac{\delta_{\upsilon \sigma }}{2\tau }\left(\Delta A^{\nu }-\tau V^{\nu }\right)\left(\Delta A^{\sigma }-\tau V^{\sigma }\right)\right]\;. \end{array}$$

The integrals can be evaluated from the known properties of a Gaussian. The integral in the first term gives $\langle \Delta A^{\mu }\rangle =\tau \delta^{\mu \nu }\frac{\partial S}{\partial A^{\nu }}$ and the integral in the second term gives $\langle \Delta A^{\mu }\Delta A^{\nu }\rangle =\tau \delta^{\mu \nu }$, so that

(43) $$\langle \Delta A^{i}\rangle =\frac{\partial A^{i}}{\partial A^{\mu }}\tau \delta^{\mu \nu }\frac{\partial S}{\partial A^{\mu }}-\frac{1}{2}\Gamma_{jk}^{i}\frac{\partial A^{j}}{\partial A^{\mu }}\frac{\partial A^{k}}{\partial A^{\nu }}\tau \delta^{\mu \nu }\;.$$

Therefore, in natural coordinates, the first two moments up to order of τ are

(44) $$\langle \Delta A^{i}\rangle =\tau g^{ij}\frac{\partial S}{\partial A^{j}}-\frac{\tau }{2}\Gamma^{i}\;,\;\;\mathrm{and}\;\;\langle \Delta A^{i}\Delta A^{j}\rangle =\tau g^{ij}\;,$$

where $\Gamma^{i}=\Gamma_{jk}^{i}g^{jk}$. Here, we see the dependence on curvature for $\langle \Delta A^{i}\rangle$ in the Christoffel symbol term. Note that it is a consequence of the dependance between $\Delta A^{i}$ and the quadratic term $\Delta A^{i}\Delta A^{j}$ in (40), which per (44) does not vanish, even for small steps. Hence, fluctuations in $A^{i}$ cannot be ignored in the ED motion, and this is the reason why the motion probes curvature. It also follows from (44) that, even in the limit τ→0, the average change $\Delta A^{i}$ does not transform covariantly.

Note that we used several words, such as “transitions”, “short step”, “continuous”, and “dynamics” without any established notion of time. In the following section, we will discuss time not as an external parameter, but as an emergent parameter from the maximum entropy transition (32) and its moments (44).

## 5. Entropic Time

Having described a short step transition, the next challenge is to study how these short steps accumulate.

### 5.1. Introducing Time

In order to introduce time, we note that $A^{\prime }$ and *A* are elements of the same manifold; therefore, $P(A^{\prime })$ and P(A) are two probability distributions over the same space. Our established solution for describing the accumulation of changes (see [28]) is to introduce a “book-keeping” parameter *t* that distinguishes the said distributions as labelled by different parameters, i.e., $P\left(A^{\prime }\right)=P_{t^{\prime }}\left(A\right)$ and $P\left(A\right)=P_{t}\left(A\right)$.

In this formalism, we will refer to these different labels as a description of the system at particular instants *t* and $t^{\prime }$. This allows us to call $P(A^{\prime }|A)$ a transition probability.

(45) $$P_{t^{\prime }}\left(A\right)=P\left(A^{\prime }\right)=\int dA\;P_{\Delta t}\left(A^{\prime }|A\right)P_{t}\left(A\right)$$

where $\Delta t=t^{\prime }-t$.

As the system changes from *A* to $A^{\prime }$ and then to $A^{\prime \prime }$. The probability $P(A^{\prime \prime })$ will be constructed from $P(A^{\prime })$, not explicitly dependent on P(A). This means that (45) represents a Markovian process: conditioned on the present $P_{t^{\prime }}\left(A\right)$, the “future” $P_{t^{\prime \prime }}\left(A\right)$ is independent of the “past” $P_{t}\left(A\right)$, where $t^{\prime \prime }>t^{\prime }>t$. It is important to notice that, under this formalism, (45) is not used to show that the process is Markovian in an existing time, but rather the concept of time that was developed here makes the dynamics Markovian by design.

It is also important to notice that the parameter *t* that is presented here is not necessarily the “physical” time (as it appears in Newton’s laws of motion or the Schrödinger equation). Our parameter *t*, which we call entropic time, is an epistemic well-ordered parameter in which the dynamics are defined.

### 5.2. The Entropic Arrow of Time

It is important to note that the marginalization process from (20) to (45) could also lead to

(46) $$P\left(A\right)=\int dA^{\prime }\;P\left(A|A^{\prime }\right)P\left(A^{\prime }\right)\;,$$

where the conditional probabilities are related by Bayes’ Theorem,

(47) $$P\left(A|A^{\prime }\right)=\frac{P(A)}{P(A^{\prime })}P\left(A^{\prime }|A\right)\;,$$

showing that a change “forward” will not happen the same way as a change “backwards” unless the system is in some form of stationary state, $P\left(A\right)=P\left(A^{\prime }\right)$. Another way to present this is that probability theory alone gives no intrinsic distinction of the change “forward” and “backward”. The fact that we assigned the change “forward” by ME implies that, in general, the change “backward” is not an entropy maximum. Therefore, the preferential direction of the flow of time arises from the entropic dynamics naturally.

### 5.3. Calibrating the Clock

One needs to define the duration Δt with respect to the motion in order to connect the entropic time to the transition probability. Time in entropic dynamics is defined so as to simplify the description of the motion. This notion of time is tailored to the system under discussion. The time interval will be chosen, so that the parameter τ that first appeared in the prior (25) takes the role of a time interval,

(48) τ=ηΔt,

where η is a constant, so that *t* has the units of time. For the remainder of this article, we will adopt η=1. In principle, any monotonic function t(τ) serves as an parameter for ordering. Our choice is a matter of convenience, as required by simplicity. Here this is implemented so that for a short transition we have the dimensionless time interval

(49) $$\Delta t=g_{ij}\langle \Delta A^{i}\Delta A^{j}\rangle \;.$$

This means that the system’s fluctuations measure the entropic time. Rather than having the changes in the system represented in terms of given time intervals (as measured by an external clock), here the system is its own clock.

The moments in (44) can be written, up to order Δt, as

(50) $$\frac{\langle \Delta A^{i}\rangle }{\Delta t}=g^{ij}\frac{\partial S}{\partial A^{j}}-\frac{1}{2}\Gamma^{i}\;,\;\;\mathrm{and}\;\;\frac{\langle \Delta A^{i}\Delta A^{j}\rangle }{\Delta t}=g^{ij}\;.$$

With this, we have established a concept of time and it is convenient to write the trajectory of the expected values in terms of a differential equation.

## 6. Diffusion and the Fokker–Planck Equation

Our goal of designing the dynamics from entropic methods is accomplished. The entropic dynamics equation of evolution is written in integral form as a Chapman–Kolmogorov Equation (45) with a transition probability given by (32). In this section, we will conveniently rewrite it in the differential form. The computed drift $\langle \Delta A^{i}\rangle$ and the fluctuation $\langle \Delta A^{i}\Delta A^{j}\rangle$ in (50) describe the dynamical process as a smooth diffusion—meaning, as defined by [63], a stochastic process in which the first two moments are calculated to the order of Δt, $\langle \Delta A^{i}\rangle =b^{i}\Delta t$, $\langle \Delta A^{i}\Delta A^{j}\rangle =\eta g^{ij}\Delta t$, and $\langle \Delta A^{i}\Delta A^{j}\Delta A^{k}\rangle =0$. Therefore, for a short transition, it is possible to write the evolution of $P_{t}\left(A\right)$, as a Fokker–Planck (diffusion) equation,

(51) $$\frac{\partial }{\partial t}P=-\frac{\partial }{\partial A^{i}}\left(Pv^{i}\right)\;,$$

where

(52) $$v^{i}=g^{ij}\frac{\partial S}{\partial A^{j}}-\frac{1}{2}g^{ij}\frac{\partial }{\partial A^{j}}\left(\log\frac{P}{g^{1/2}}\right)\;.$$

The derivation of (51) and (52) takes into account the fact that the space in which the diffusion happens is curved and it is given in Appendix B. In equation (52), we see that the current velocity $v^{i}$ consists of two components. The first term is the drift velocity that is guided by the entropy gradient and the second term is an osmotic velocity, which is a term that is driven by differences in probability density. The examples that are presented in the following section will show how these terms interact and the dynamical properties that are derived from each.

### Derivatives and Divergence

Because the entropy *S* is a scalar, the velocity that is defined in (52) is a contravariant vector. However, (51) is not a manifestly invariant equation. To check its consistency, it is convenient to write it in terms of the invariant object *p*, being defined as

(53) $$p\left(A\right)=\frac{P(A)}{g^{1/2}\left(A\right)}\;,$$

meaning that *p* is the probability of *A* divided by the volume element, in terms of which (51) becomes

(54) $$\frac{\partial }{\partial t}p=-\frac{1}{g^{1/2}}\frac{\partial }{\partial A^{i}}\left(g^{1/2}\;pv^{i}\right)\;.$$

We can recognize, on the right-hand side, the covariant divergence of the contravariant vector $pv^{i}$, which can be written in the manifestly covariant form

(55) $$\frac{\partial }{\partial t}p=-D_{i}\left(pv^{i}\right)\;,$$

where $D_{i}$ is the covariant derivative. The fact that the covariant derivative arises from the dynamical process is the direct indication that even when evolving the invariant object *p* the curvature of the space is taken into account. We can identify (55) as a continuity equation—generalized to the parallel transport in a curved space, as evidenced by the covariant divergence—where the flux, $j^{i}=pv^{i}$, can be written from (52) and (53) as

(56) $$j^{i}=pg^{ij}\frac{\partial S}{\partial A^{j}}-\frac{1}{2}g^{ij}\frac{\partial p}{\partial A^{j}}\;.$$

The second term, which is related to the osmotic velocity, is a Fick’s law with diffusion tensor $D^{ij}=g^{ij}/2$. Note that this is identified from purely probabilistic arguments, rather than assuming a repulsive interaction from the microstate dynamics.

Having the dynamics fully described, we can now study its consequences, as will be done in the following section.

## 7. Examples

We established the entropic dynamics by finding the transition probability (32), presenting it as a differential equation in (51), (52), and presenting it as the invariant Equation (55). We want to show some examples of how it would be applied and what are the results achieved. Our present goal is not to search for realistic models, but to search for models that are both mathematically simple and general enough so it can give insight on how to use the formalism.

We will be particularly interested in two properties: the drift velocity,

(57) $$v_{D}^{i}=g^{ij}\frac{\partial S}{\partial A^{j}}\;,$$

which is the first term in (52), and the static states, $v^{i}=0$, which are a particular subset of the dynamical system’s equilibrium $\partial_{t}P=0$. These are obtained from (52) as

(58) $$v^{i}=0\Rightarrow \frac{\partial S}{\partial A^{i}}-\frac{1}{2}\frac{\partial }{\partial A^{i}}\log\left(\frac{P}{g^{1/2}}\right)=0$$

allowing for one to write the static probability

(59) $$P\left(A\right)\propto g^{1/2}\left(A\right)\exp\left[2S\left(A\right)\right]\;,$$

where the factor of 2 in the exponent comes from the diffusion tensor $D^{ij}=g^{ij}/2$ that is explained in Section 6. This result shows that the invariant stationary probability density (53) is

(60) p(A)∝exp[2S(A)].

### 7.1. A Gaussian Manifold

The statistical manifold defined by the mean values and correlations of a random variable, the microstate *x*, is the space of Gaussian distribution, which is an example of a canonical distribution. Here, we consider the dynamics of a two-dimensional spherically symmetric Gaussian with a non-uniform variance, $\sigma \left(A\right)=\sigma \left(A^{1},A^{2}\right)$, as defined by

(61) $$x^{1}=A^{1},x^{2}=A^{2},\mathrm{and}\left(x^{i}-A^{i}\right)\left(x^{j}-A^{j}\right)=\sigma^{2}\left(A\right)\delta^{ij}.$$

These Gaussians are of the form,

(62) $$\rho \left(x|A\right)=\frac{1}{2\pi \sigma^{2}\left(A\right)}\exp\left(-\frac{1}{2\sigma^{2}\left(A\right)}\sum_{i=1}^{2}{\left(x^{i}-A^{i}\right)}^{2}\right)$$

The entropy of (62) relative to a uniform background measure is given by

(63) $$S\left(A\right)=\log(2\pi \sigma {\left(A\right)}^{2})$$

The space of Gaussians with a uniform variance, σ(A)= constant, is flat and the dynamics turn out to be a rather trivial spherically symmetric diffusion. Choosing the variance to be non-uniform yields richer and more interesting dynamics. Because this example is pursued for purely illustrative purposes, we restrict to two dimensions and spherically symmetric Gaussians. The generalization is immediate.

The FRIM for a Gaussian distribution is found using (12) (see also [13]), to be

(64) $$dl^{2}=\frac{4}{\sigma^{2}}{\left(d\sigma \right)}^{2}+\frac{\delta_{ij}}{\sigma^{2}}dA^{i}dA^{j}\;,$$

so that, using

(65) $$d\sigma =\frac{\partial \sigma }{\partial A^{i}}dA^{i}\;,$$

the induced metric $dl^{2}=g_{ij}dA^{i}dA^{j}$ leads to

(66) $$g_{ij}=\frac{1}{\sigma^{2}}\left(4\frac{\partial \sigma }{\partial A^{i}}\frac{\partial \sigma }{\partial A^{j}}+\delta_{ij}\right)\;.$$

#### Gaussian Submanifold around an Entropy Maximum

We present an example of our dynamical model that illustrates the motion around an entropy maximum. A simple way to manifest it is to recognize that, in (52), −S plays a role analogous to a potential. A rotationally symmetric quadratic potential can then be sustituted in (63), leading to

(67) $$\sigma \left(A\right)=\exp\left(-\frac{{\left(A^{1}\right)}^{2}+{\left(A^{2}\right)}^{2}}{4}\right)\;,$$

which, substituted in (66), yields the metric

(68) $$g_{ij}=\left[\begin{array}{cc} {\left(A^{1}\right)}^{2}+\sigma^{-2} & A^{1}A^{2}\\ A^{1}A^{2} & {\left(A^{2}\right)}^{2}+\sigma^{-2} \end{array}\right]\;,$$

so that

(69) $$g^{1/2}=\sqrt{\left[{\left(A^{1}\right)}^{2}+{\left(A^{2}\right)}^{2}\right]\sigma^{-2}+\sigma^{-4}}\;.$$

The scalar curvature for the Gaussian submanifold can be calculated from (68) as

(70) $$R=\frac{\phi^{2}-2\phi }{{\left(\phi^{2}+\sigma^{-2}\right)}^{2}}\sigma^{2},\mathrm{where}\;\phi ={\left(A^{1}\right)}^{2}+{\left(A^{2}\right)}^{2}\;.$$

**Figure 1:**
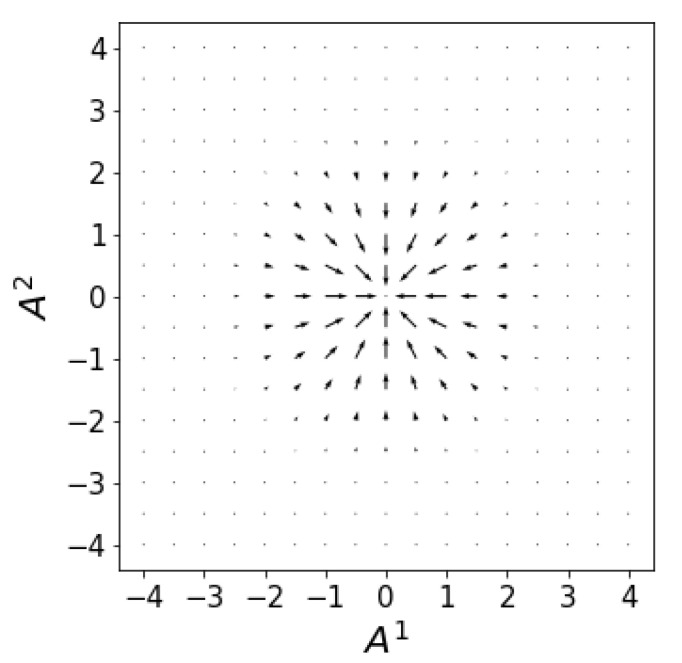
The drift velocity field (71) drives the flux along the entropy gradient.

**Figure 2:**
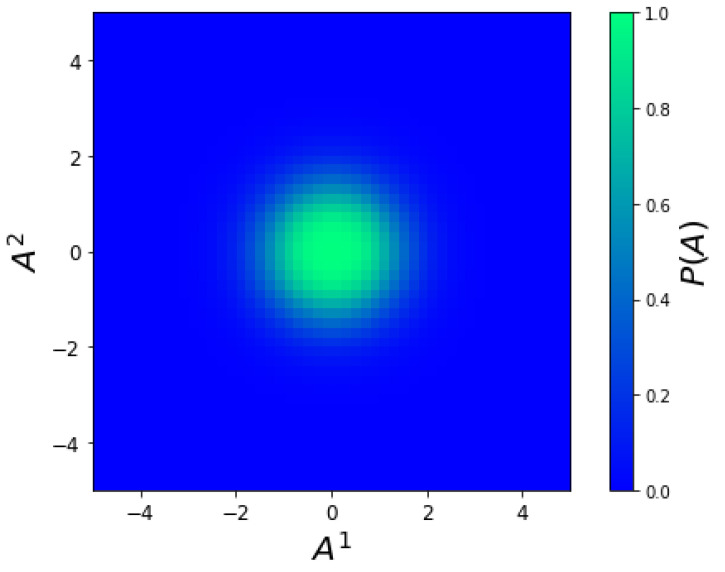
Equilibrium stationary probability (72).

From (57), the drift velocity (Figure 1) is

(71) $$v_{D}^{1}=-\frac{A^{1}\sigma^{-2}}{g}\mathrm{and}v_{D}^{2}=-\frac{A^{2}\sigma^{-2}}{g}\;.$$

and, from (59), the static probability (Figure 2) is

(72) $$P\left(A\right)\propto 4\pi^{2}g^{1/2}\sigma^{4}\;.$$

The static distribution results from the dynamical equilibrium between two opposite tendencies. One is the drift velocity field that drives the distribution along the entropy gradient towards the entropy maximum at the origin. The other is the osmotic diffusive force that we identified earlier as the ED analogue of Fick’s law. This osmotic force drives the distribution against the direction of the probability gradient and prevents it from becoming infinitely concentrated at the origin. At equilibrium, the cancellation between these two opposing forces results in the Gaussian distribution, Equation (72).

### 7.2. 2-Simplex Manifold

Here, we discuss an example of discrete microstates. The macrostate coordinates, being expected values, are continuous variables. Our subject matter will be a three-state system, x∈{1,2,3}, such as, for example, a 3-sided die. The statistical manifold is the 2-dimensional simplex and the natural coordinates are the probabilities themselves,

(73) $$S_{2}=\rho \left(x\right)|\rho \left(x\right)\geq 0\;,\;\sum_{x=1}^{3}\rho \left(x\right)=1.$$

The distributions on the two-simplex are Gibbs distributions defined by the sufficient statistics of functions

(74) $$a^{i}\left(x\right)=\delta_{x}^{i}\;\mathrm{so}\;\mathrm{that}\;A^{i}=\langle a^{i}\rangle =\rho \left(i\right)\;.$$

The entropy relative to the uniform discrete measure is

(75) $$S=-\sum_{i=1}^{3}\rho \left(i\right)\log\left(\rho \left(i\right)\right)=-\sum_{i=1}^{3}A^{i}\log\left(A^{i}\right)\;,$$

and the information metric is given by

(76) $$g_{ij}=\sum_{k=1}^{3}\rho^{k}\frac{\partial \log(\rho^{k})}{\partial A^{i}}\frac{\partial \log(\rho^{k})}{\partial A^{j}}\;.$$

The two-simplex arises naturally from probability theory due to normalization when one identifies the macrostate of interest to be the probabilities themselves. The choice of sufficient statistics (74) implies that the manifold is a two-dimensional surface, since, due to the normalization, one can write $A^{3}=1-A^{1}-A^{2}$. We will use the the tuple $\left(A^{1},A^{2}\right)$ as our coordinates and $A^{3}$ as a function of them. In this scenario, one finds a metric tensor

(77) $$g_{ij}=\left[\begin{array}{cc} \frac{1}{A^{3}}+\frac{1}{A^{1}} & \frac{1}{A^{3}}\\ \frac{1}{A^{3}} & \frac{1}{A^{3}}+\frac{1}{A^{2}} \end{array}\right]\;,$$

which induces the volume element

(78) $$g^{1/2}=\sqrt{\frac{1}{A^{1}A^{2}A^{3}}}\;.$$

**Figure 3:**
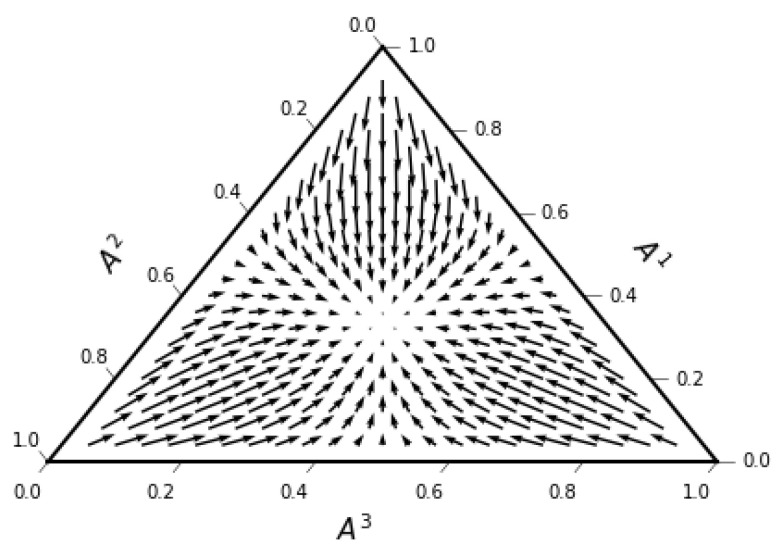
Drift velocity field for the two-simplex in (79). The ternary plots ware created using python-ternary library [64].

As is well known, the simplex is characterized by a constant curvature R=1/2; the two-simplex is the positive octant of a sphere. From (57), the drift velocity (Figure 3) is

(79) $$\begin{array}{cc} v_{D}^{1} & =A^{1}\left[A^{2}\log\left(\frac{A^{2}}{A^{3}}\right)+\left(A^{1}-1\right)\log\left(\frac{A^{1}}{A^{3}}\right)\right]\\ v_{D}^{2} & =A^{2}\left[A^{1}\log\left(\frac{A^{1}}{A^{3}}\right)+\left(A^{2}-1\right)\log\left(\frac{A^{2}}{A^{3}}\right)\right]\;, \end{array}$$

Additionally, the static probability is

(80) $$P\left(A\right)\propto g^{1/2}\prod_{i=1}^{3}{\left(A^{i}\right)}^{-2A^{i}}\;.$$

From the determinant of the metric, we note that the static probability (80) diverges at the boundary of the two-simplex. This reflects the fact that a two-state system (say, i=1,2) is easily distinguishable from a three-state system (i=1,2,3). Indeed, a single datum i=3 will tell us that we are dealing with a three-state system.

**Figure 4:**
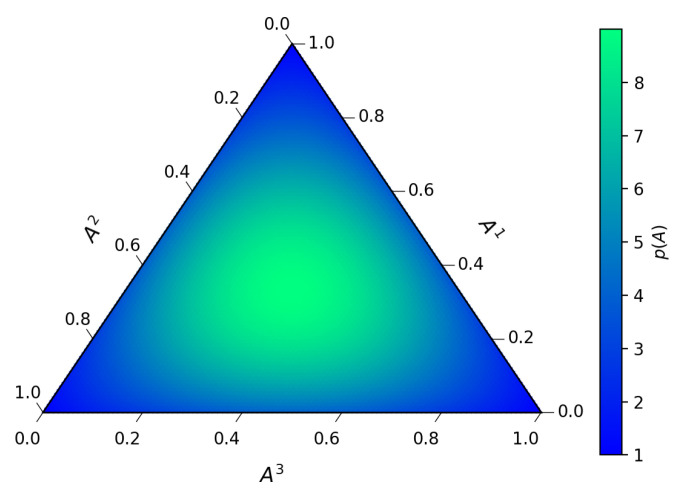
Static invariant stationary probability for the three-state system.

On the other hand, we can see (Figure 4) that this divergence is not present in the invariant stationary probability (53).

As in the Gaussian case discussed in the previous section, the static equilibrium results from the cancellation of two opposing forces: the entropic force along the drift velocity field towards the center of the simplex is cancelled by the osmotic diffusive force away from the center.

## 8. Conclusions

We conclude with a summary of the main results. In this paper, the entropic dynamics framework has been extended to describe dynamics on a statistical manifold. ME played an instrumental role in that it allowed us to impose constraints that are not in the standard form of expected values.

The resulting dynamics, which follow from purely entropic considerations, take the form of a diffusive process on a curved space. The effects of curvature turn out to be significant. We found that the probability flux is the result of two components. One describes a flux along the entropy gradient and the other is a diffusive or osmotic component that turns out to be the curved-space analogue of Fick’s law with a diffusion tensor $D^{ij}=g^{ij}/2$ that is given by information geometry.

A highlight of the model is that it includes an “entropic” notion of time that is tailored to the system under study; the system is its own clock. This opens the door to the introduction of a notion of time that transcends physics and it might be useful for social and ecological systems. The emerging notion of entropic time is intrinsically directional. There is a natural arrow of time that manifests itself in a simple description of the approach to equilibrium.

The model developed here is rather minimal in the sense that the dynamics could be extended by taking additional relevant information into account. For example, it is rather straightforward to enrich the dynamics by imposing additional constraints

(81) $$\langle \Delta A^{i}\rangle F_{i}\left(A\right)=\kappa^{\prime }\;,$$

involving system-specific functions $F_{i}\left(A\right)$ that carry information regarding correlations. This is the kind of further developments that we envisage in future work.

As illustrative examples, the dynamics were applied to two general spaces of probability distributions. A submanifold of the space of two-dimensional Gaussians and the space of probability distributions for a three-state system (two-simplex). In each of these, we were able to provide insight on the dynamics by presenting the drift velocity (57) and the equilibrium stationary states (59). Additionally, as future work, we intend to apply the dynamics developed here in the distributions found in network sciences [65].

## Figures and Tables

**Table 1:** Identification of sufficient statistics, priors and Lagrange multipliers for some well-known probability distributions.

Distribution	λ Parameter	Suff. Stat.	Prior
Exponent Polynomial $\rho \left(x|\beta \right)=\frac{\sqrt[{k}]{\beta }}{\Gamma (1+1/\beta )}e^{-\beta x^{k}}\;$	λ=β	$a\left(x\right)=x^{k}$	uniform
Gaussian $\rho \left(x|\mu ,\sigma \right)=\frac{1}{\sqrt{2\pi \sigma^{2}}}\exp\left[-\frac{{\left(x-\mu \right)}^{2}}{2\sigma^{2}}\right]\;\;$	$\lambda =\left(-\frac{\mu }{\sigma^{2}}\;,\frac{1}{2\sigma^{2}}\right)$	$a\left(x\right)=\left(x,x^{2}\right)$	uniform
Multinomial (k) $\rho \left(x|\theta \right)=\frac{n!}{x_{1}!\ldots x_{k}!}\theta_{1}^{x_{1}}\ldots \theta_{k}^{x_{k}}$	$\lambda =-\log(\theta_{1},\theta_{2},\ldots ,\theta_{k})\;$	$a=(x_{1},\ldots x_{k})\;$	$q\left(x\right)=\prod_{i=1}^{k}x_{i}!$
Poisson $\rho \left(x|m\right)=\frac{m^{x}}{x!}e^{-m}$	λ=−logm	a(x)=x	q(x)=1/x!
Mixed power laws $\rho \left(x|\alpha ,\beta \right)=\frac{x^{-\alpha }e^{-\beta x}}{\beta^{\alpha -1}\Gamma \left(1-\alpha \right)}$	λ=(α,β)	a=(logx,x)	uniform

## Data Availability

No new data were created or analyzed in this study. Data sharing is not applicable to this article. Code producing the graphs in Figure 1, Figure 2, Figure 3 and Figure 4 is available upon request.

## Abbreviations

The following abbreviations are used in this manuscript:

MaxEnt	MAXimum ENTropy—according to Jaynes [2]
ME	(Modern) Maximum Entropy—since Shore & Johnson [7]
FRIM	Fisher-Rao Information Metric
ED	Entropic Dynamics

## Appendix A. Obtaining the Prior

In this appendix we derive the prior transition probability from *A* to $A^{\prime }$ seen in (25). This is achieved by maximizing the entropy

(A1) $$S\left[Q\right]=-\int dA^{\prime }\;Q\left(A^{\prime }|x,A\right)\log\left(\frac{Q(A^{\prime }|x,A)}{R(A^{\prime }|x,A)}\right)\;,$$

where $R(A^{\prime }|x,A)$, the prior for (A1), encodes information about an unbiased transition of the systems. The posterior of (A1), $Q(A^{\prime }|x,A)$, becomes the prior in (21).

At this stage *A* could evolve into any $A^{\prime }$ and the only assumption is that the assigned prior for (A1) leads to equal probabilities for equal volumes; thus, ignoring biases. That is achieved by a prior proportional to the volume element $R\left(A^{\prime }|x,A\right)\;\propto \;g^{1/2}\left(A^{\prime }\right)$, where $g\left(A\right)=\det g_{ij}\left(A\right)$. There is no need to address the normalization of *R* since it will have no effect on the posterior.

The chosen constraint represents an isotropic and continuous motion on the manifold. This will be imposed by

(A2) $$\int dA^{\prime }\;Q\left(A^{\prime }|x,A\right)\;g_{ij}\Delta A^{i}\Delta A^{j}=K\;.$$

where *K* is a small quantity, since $g_{ij}\Delta A^{i}\Delta A^{j}$ is invariant only in the limit for short steps $\Delta A^{i}\to 0$. Therefore, eventually, K→0.

The result of maximizing (A1) under (A2) and normalization is

(A3) $$Q\left(A^{\prime }|x,A\right)\propto g^{1/2}\left(A^{\prime }\right)\exp\left(-\alpha \;g_{ij}\Delta A^{i}\Delta A^{j}\right)\;,$$

where α is the Lagrange multiplier associated to (A2). As the result requires K→0 to make it geometrically invariant, the conjugated Lagrange multiplier should be allowed to be taken to infinity. This allows us to define τ=1/α, such that the short step limit will lead to τ→0.

Note that, since no motion in *x* and no correlation between *x* and $A^{\prime }$ is induced by the constraints, the result does not depend on the previous microstate *x*, $Q\left(A^{\prime }|x,A\right)=Q\left(A^{\prime }|A\right)$.

## Appendix B. Derivation of the Fokker-Planck Equation

The goal of this appendix is to show that for a dynamics that is a smooth diffusion in a curved space, can be written as a Fokker-Planck equation and to obtain its velocity (52) from the moments for the motion (50). In order to do so, it is convenient to define a drift velocity

(A4) $$b^{i}=\lim_{\Delta t\to 0}\frac{\langle \Delta A^{i}\rangle }{\Delta t}=g^{ij}\frac{\partial S}{\partial A^{j}}-\frac{1}{2}\Gamma^{i}\;.$$

First, let us analyze the change of a smooth integrable function f(A) as the system transitions from *A* to $A^{\prime }$. A smooth change in the function f(A) will be

(A5) $$\Delta f\left(A\right)=\frac{\partial f}{\partial A^{i}}\Delta A^{i}+\frac{1}{2}\frac{\partial^{2}f}{\partial A^{i}\partial A^{j}}\Delta A^{i}\Delta A^{j}+o\left(\Delta t\right)\;,$$

since a cubic term, $\Delta A^{i}\Delta A^{j}\Delta A^{k}$ would be o(Δt). In a smooth diffusion we can take the expected value of (A5) with respect to $P(A^{\prime }|A)$ as

(A6) $$\langle \Delta f\left(A\right)\rangle =\int dA^{\prime }\;P\left(A^{\prime }|A\right)\left(f\left(A^{\prime }\right)-f\left(A\right)\right)=\left(b^{i}\frac{\partial }{\partial A^{i}}+\frac{1}{2}g^{ij}\frac{\partial^{2}}{\partial A^{i}\partial A^{j}}\right)f\left(A\right)\Delta t\;.$$

which can be further averaged in P(A). The left-hand side will be

(A7) $$\begin{array}{cc} \int dA\;P\left(A\right)\int dA^{\prime }\;P\left(A^{\prime }|A\right)\left(f\left(A^{\prime }\right)-f\left(A\right)\right) & =\int dAdA^{\prime }P\left(A^{\prime },A\right)\left(f\left(A^{\prime }\right)-f\left(A\right)\right) \end{array}$$

(A8) $$\begin{array}{cc} \; & =\int dA^{\prime }\;P\left(A^{\prime }\right)f\left(A^{\prime }\right)-\int dA\;P\left(A\right)f\left(A\right) \end{array}$$

while the right hand is

(A9) $$\int dA\;P\left(A\right)\left(b^{i}\frac{\partial }{\partial A^{i}}+\frac{1}{2}g^{ij}\frac{\partial^{2}}{\partial A^{i}\partial A^{j}}\right)f\left(A\right)\Delta t\;.$$

such that they equate to

(A10) $$\int dA^{\prime }\;P\left(A^{\prime }\right)f\left(A^{\prime }\right)-\int dA\;P\left(A\right)f\left(A\right)=\int dA\;P\left(A\right)\left(b^{i}\frac{\partial }{\partial A^{i}}+\frac{1}{2}g^{ij}\frac{\partial^{2}}{\partial A^{i}\partial A^{j}}\right)f\left(A\right)\Delta t\;.$$

As established in Section 5, P(A) and $P(A^{\prime })$ are distributions at the instants *t* and $t^{\prime }$ respectively.

(A11) $$\int dA\;\left(\frac{P_{t^{\prime }}\left(A\right)-P_{t}\left(A\right)}{\Delta t}\right)f\left(A\right)=\int dA\;P\left(A\right)\left(b^{i}\frac{\partial }{\partial A^{i}}+\frac{1}{2}g^{ij}\frac{\partial^{2}}{\partial A^{i}\partial A^{j}}\right)f\left(A\right)\Delta t\;,$$

which can be partially integrated in the limit of small steps

(A12) $$\int dA\;\left(\frac{\partial P(A)}{\partial t}\right)f\left(A\right)=\int dA\;\left(-\frac{\partial }{\partial A^{i}}\left(b^{i}P\left(A\right)\right)+\frac{1}{2}\frac{\partial^{2}}{\partial A^{i}\partial A^{j}}\left(g^{ij}P\left(A\right)\right)\right)f\left(A\right)\;.$$

Due to the generality of *f* as test function, we identify the integrants,

(A13) $$\frac{\partial }{\partial t}P\left(A\right)=-\frac{\partial }{\partial A^{i}}\left(b^{i}P\left(A\right)-\frac{1}{2}\frac{\partial }{\partial A^{j}}\left(g^{ij}P\left(A\right)\right)\right)\;,$$

and substitute $b^{i}$ (A4) for general coordinates,

(A14) $$\frac{\partial }{\partial t}P\left(A\right)=-\frac{\partial }{\partial A^{i}}\left(g^{ij}\frac{\partial S}{\partial A^{j}}P\left(A\right)-\frac{1}{2}\Gamma^{i}P\left(A\right)-\frac{1}{2}\left(\frac{\partial g^{ij}}{\partial A^{j}}\right)P\left(A\right)-\frac{1}{2}g^{ij}\frac{\partial P(A)}{\partial A^{j}}\right)\;,$$

and the contracted Christoffel symbols can be substituted in the identity

(A15) $$\Gamma^{i}=-\frac{1}{g^{1/2}}\frac{\partial }{\partial A^{j}}\left(g^{1/2}g^{ij}\right)=-\frac{\partial g^{ij}}{\partial A^{j}}-g^{ij}\frac{\partial \log g^{1/2}}{\partial A^{j}}\;.$$

Here we see that the effect of curvature—encoded by the Christoffel symbols—substitute in the differential Equation (A13) obtaining

(A16) $$\frac{\partial }{\partial t}P\left(A\right)=-\frac{\partial }{\partial A^{i}}\left(g^{ij}\frac{\partial S}{\partial A^{j}}-\frac{1}{2}g^{ij}\frac{\partial }{\partial A^{j}}\left(\log\frac{P(A)}{g^{1/2}}\right)\right)P\left(A\right)\;,$$

where the second term inside the parenthesis above is the result of taking the curvature into account. The result is a Fokker-Planck equation that is usefully written in the continuity form

(A17) $$\frac{\partial }{\partial t}P=-\frac{\partial }{\partial A^{i}}\left(Pv^{i}\right)\;,$$

where

(A18) $$v^{i}=g^{ij}\frac{\partial S}{\partial A^{j}}-\frac{1}{2}g^{ij}\frac{\partial }{\partial A^{j}}\left(\log\frac{P}{g^{1/2}}\right)\;,$$

completing the derivation.
